# The viral hypothesis in Alzheimer’s disease: SARS-CoV-2 on the cusp

**DOI:** 10.3389/fnagi.2023.1129640

**Published:** 2023-03-15

**Authors:** Nanyang Liu, Xuefan Jiang, Hao Li

**Affiliations:** ^1^Xiyuan Hospital, China Academy of Chinese Medical Sciences, Beijing, China; ^2^Graduate School, Beijing University of Chinese Medicine, Beijing, China; ^3^Wangjing Hospital, China Academy of Chinese Medical Sciences, Beijing, China

**Keywords:** Alzheimer’s disease, SARS-CoV-2, COVID-19, viral hypothesis, neuroinflammation

## Abstract

Increasing evidence highlights that infection with severe acute respiratory syndrome coronavirus 2 (SARS-CoV-2) has long-term effects on cognitive function, which may cause neurodegenerative diseases like Alzheimer’s disease (AD) in the future. We performed an analysis of a possible link between SARS-CoV-2 infection and AD risk and proposed several hypotheses for its possible mechanism, including systemic inflammation, neuroinflammation, vascular endothelial injury, direct viral infection, and abnormal amyloid precursor protein metabolism. The purpose of this review is to highlight the impact of infection with SASR-CoV-2 on the future risk of AD, to provide recommendations on medical strategies during the pandemic, and to propose strategies to address the risk of AD induced by SASR-CoV-2. We call for the establishment of a follow-up system for survivors to help researchers better understand the occurrence, natural history, and optimal management of SARS-CoV-2-related AD and prepare for the future.

## Introduction

Alzheimer’s disease (AD) is a common age-related neurodegenerative disease with clinical manifestations that include a gradual decline in memory, language, judgment, and loss of self-care ability as the disease progresses. The most characteristic pathological manifestations of AD are abnormal deposition of amyloid-beta (Aβ) peptide plaques and tau hyperphosphorylation, which form senile plaques and neurofibrillary tangles (NFTs) in neurons, respectively (Jia et al., 2020). This incurable disease is expected to affect approximately 100 million people worldwide by 2050 (Alzheimer’s Dementia, 2020). The etiology is multifactorial, with the possible inclusion of neuroinflammation, oxidative stress, intestinal flora, synaptic plasticity, vascular abnormalities, and infectious agents (Long and Holtzman, 2019). Although approximately 25% of cases are familial, approximately 75% of AD cases have an unknown disease background.

**Figure 1:**
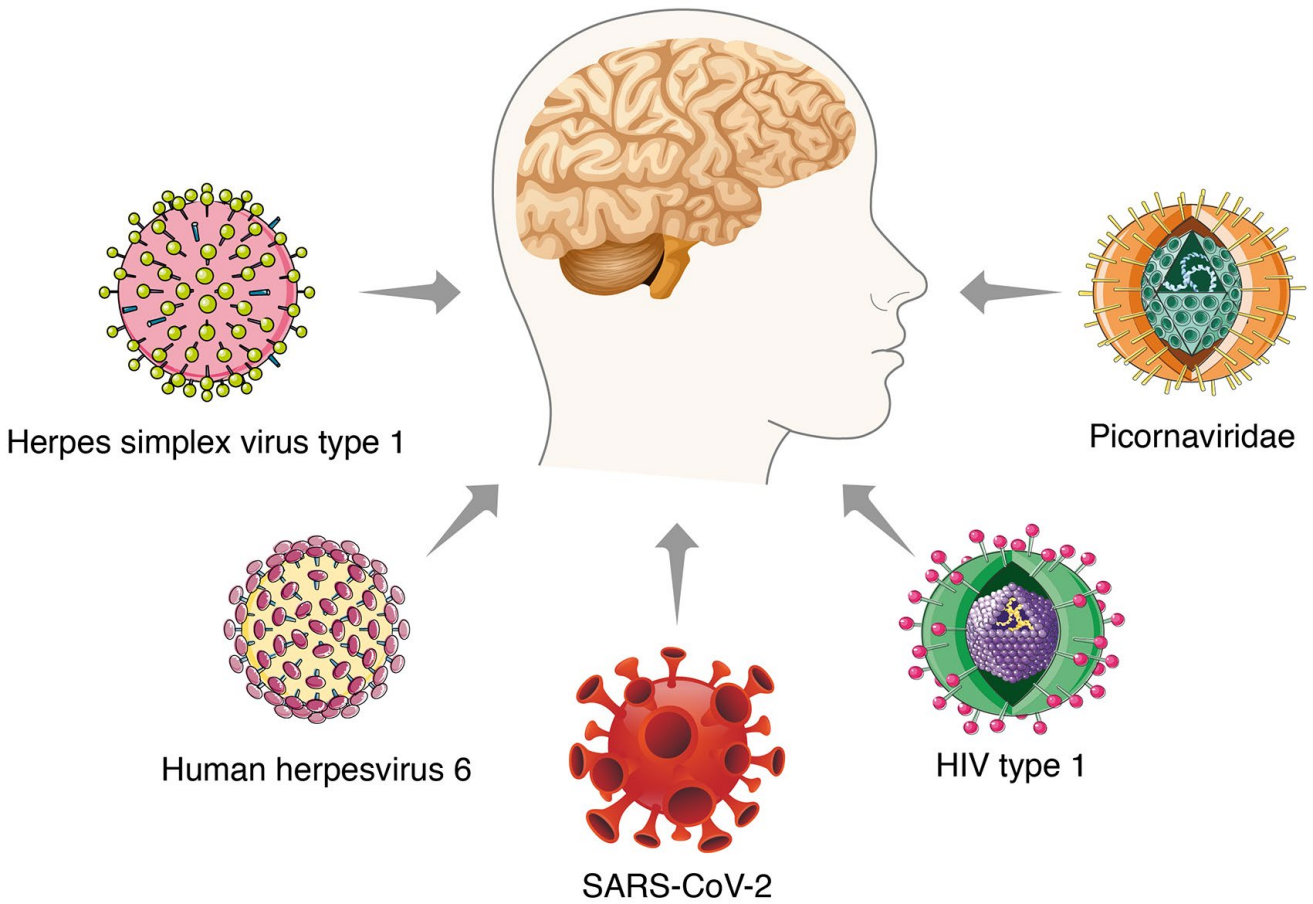
Several viruses associated with Alzheimer’s disease.

Most studies revealed the unique roles of Aβ and tau proteins in AD pathogenesis. Despite enormous work, no effective strategies have emerged. It is necessary to explain the pathogenesis from another point of view and look for possible treatments. The inflammation-pathogen infection hypothesis has been accepted as a substitute for the amyloid hypothesis in the past few years. Bacteria and viruses, as well as several other infectious factors, are considered to be related to the pathogenesis of AD (Lopatko Lindman et al., 2021; Figure 1). Notably, multiple excellent studies have steadily revealed the potential role of several viruses in AD pathology, such as herpes simplex virus type 1 (HSV1), herpes simplex virus type 2 (HSV2), measles virus, adenoviruses, cytomegalovirus, poliovirus, hepatitis B virus, and influenza virus (Wang et al., 2022). These works support the virus hypothesis and the potential of antiviral treatment to overcome AD.

The widespread Corona Virus Disease 2019 (COVID-19) epidemic pushed the viral hypothesis to neurologists. Patients with COVID-19 experience frequent neurological symptoms, such as brain fog, headaches, cognitive impairment, and chemosensory disturbances (Wang et al., 2022), and survivors have complained of long-lasting neurological sequelae. Autopsies of patients who died of COVID-19 found traces of severe acute respiratory syndrome coronavirus 2 (SARS-CoV-2) infection in the brain (Barbieri et al., 2022), which suggests that it invades and causes irreversible damage to brain tissue, and it may predispose survivors to neurodegenerative diseases. Herein, we focus on the possibility that SARS-CoV-2 infection increases the risk of developing AD and highlight several mechanisms. Although there is no direct evidence to support the association between SARS-CoV-2 and the pathological mechanism of AD, we emphasize this possibility because cytokine storms and high levels of inflammation inside the brain likely have long-term neuropsychiatric consequences.

### Viral hypothesis in Alzheimer’s disease

The idea that microbial infections trigger neurodegenerative diseases has been around since the 1950s. At the time scientists speculated that acute viral infections could lead to progressive neurological damage decades later (Ball, 1986). Although evidence for this association has been mounting, the mechanism by which the virus causes neurodegeneration remains hypothetical. A common hypothesis is that viral infection triggers an abnormal immune response that persists for years and eventually produces neurological damage associated with certain brain diseases (Ball, 1982). Evidence that viral infection plays a role in AD has long been investigated. Some viruses that cause lifelong persistent infection are upregulated in the central nervous system (CNS) of AD patients and are associated with pathology (Wang et al., 2022). Studies have found that approximately 25% of AIDS patients who did not receive combined antiretroviral therapy will develop neurological disorders associated with Aβ plaque deposition and tau protein aggregation (Hategan et al., 2019). Viral gene products are directly neurotoxic or indirectly trigger neuroinflammatory processes (Lannuzel et al., 1997; Li et al., 2015). Viral infection also affects the processing, deposition, and clearance of AD-related proteins (Kodidela et al., 2019).

The herpes virus is one of the most concerned agents in the viral infection hypothesis. Numerous studies have shown that individuals with a history of herpes virus infection are more likely to develop AD in later life than the general population (Steel and Eslick, 2015). Herpes simplex virus type 1 (HSV-1) is a neurotropic double-stranded DNA virus that infects peripheral sensory neurons and is permanently latent in the trigeminal ganglia (Ball et al., 2001). Occasionally, latent HSV-1 is reactivated by nonspecific inflammation and migrates to the trigeminal nucleus located in the brainstem. It eventually reaches the thalamus and sensory cortex and results in devastating viral encephalitis or persistent latent infection of the central nervous system (Lewandowski et al., 2002; Li Puma et al., 2019). Pioneering research by Sequiera et al. (1979) identified the HSV-1 genome in brain samples from AD patients. Since then, numerous groups have used different techniques to study the relationship between dementia and HSV-1 in brain samples and have progressively identified HSV-1 as a potential factor associated with sporadic AD. Most of these studies demonstrated that repeated HSV-1 reactivation induced Aβ accumulation and increased levels of phosphorylated tau in the brain (Martin et al., 2014; de Chiara et al., 2019). However, not every individual with HSV-1 will develop AD, which may be due in part to environment, genetics, and comorbidities. Linard et al. (2020) recently assessed the impact of HSV-1 infection on AD incidence based on a genetic susceptibility factor, apolipoprotein E (APOE). The author found that HSV1 reactivation was more frequent in APOE4 carriers than in APOE4-negative subjects, which suggests that HSV1 has a higher risk of developing AD in subjects with the APOE4 allele (Linard et al., 2020).

Human herpesvirus 6 (HHV6) belongs to the β herpesvirus subfamily, which includes two distinct species. It infects nerve cells and is associated with a variety of neurological diseases. HHV6 uses the olfactory pathway as a route to the CNS (Harberts et al., 2011). HHV6A infection may be associated with AD (Tang et al., 2022). Human herpesvirus 6 is frequently detected in AD and healthy older brains. Westman et al. (2017) showed that AD subjects had significantly reduced HHV6 IgG reactivity compared to normal controls. Although these data suggest an association of HHV6 with AD pathogenesis, these findings may represent a causal relationship or reflect an opportunistic passenger in neurodegeneration. To address this problem, Readhead et al. (2018) constructed a multiscale network of late-onset AD-associated viromes and integrated genomic, transcriptomic, proteomic, and histopathological data from four brain regions in human post-mortem tissue. They observed increases in HHV6A and human herpesvirus 7 in AD patients compared to controls. These results were replicated in two additional, independent, geographically dispersed cohorts. Notably, they observed a regulatory relationship between viral abundance and regulators of APP metabolism, including HHV6A-induced expression of APBB2, APPBP2, BIN1, BACE1, CLU, PICALM, and PSEN1. This study provides compelling evidence that specific viral species contribute to the development of neuropathology and AD. However, the current evidence is conflicting. A recent study using RNA-sequencing datasets and DNA samples from AD and non-AD control brains did not show an association between HHV6 and AD (Allnutt et al., 2020). There were no statistically significant differences in HHV6 seroprevalence, antibody titres or affinity between the three groups (Agostini et al., 2016). These inconsistent views may be due to differences in assay methods and sample sizes used. Despite the controversy, HHV6 is detected in a substantial proportion of AD patients. However, it does not appear to be an independent cause of AD but may act in conjunction with other risk factors, such as the APOE4 allele.

Picornaviridae are the most widespread disease of all viral families. Infections may be asymptomatic or lead to clinical syndromes, such as the common cold, febrile rash illness, conjunctivitis, hepatitis, myositis, and myocarditis. Picornaviruses infect the CNS, destroy pyramidal neurons in the hippocampus and cause various neurological symptoms, such as meningoencephalitis and myelitis. Animal models infected with picornaviruses exhibit memory impairment, which is associated with hippocampal damage (Buenz et al., 2006). They found LV viral infection in AD patients but not in controls. Notably, numerous amyloid plaques were detected at the site of LV-infected limbic cortical tissue damage (Niklasson et al., 2020).

These studies are the most discussed viruses related to AD pathology. Other candidates, such as Borna disease virus, HIV type 1 and JC virus, are reported sporadically. These works provide some information about the pathogenic role of viral infection. The underlying mechanisms primarily involve long-term immune system activation and persistent neuroinflammation caused by viral infection. However, most correlation studies were largely indeterminate of causality. Collectively, previous studies have highlighted (1) Aβ may act as a protective mechanism to capture viruses, (2) viruses trigger AD risk through a series of signaling pathways in human brain tissue, and (3) APOE4 increases the effect of viral reactivation on AD risk. Although the viral infection hypothesis is largely correlated rather than causation, we cannot ignore this possibility, especially when COVID-19 survivors complain of persistent memory problems.

### Neurological manifestations of SARS-CoV-2

The novel coronavirus disease, which broke out at the end of 2019, was caused by SARS-CoV-2 and has been raging worldwide for 2 years (Liu et al., 2021). Unfortunately, there is still no suitable strategy to stop the viral spread. The world’s attention initially focused on lung function as the most vulnerable organ. Over time, an extremely wide range of neurological symptoms has been reported in patients and survivors. While much remains to be learned about the effects of COVID-19 on the brain, it is worth considering that infection may increase the risk of neurological disease based on the overlap of neurological and immunological alterations. Mao and colleagues recently performed a retrospective observational case series focusing on neurological manifestations in hospitalized patients with COVID-19 (Mao et al., 2020; Zhou et al., 2020). Neurological manifestations were present in approximately 36.4% of the 214 patients, which was the starting point for evidence of neurological complications following infection with COVID-19. An increasing number of studies subsequently reported neurological complications of COVID-19, including chemosensory disturbances, seizures, dizziness, and headaches (Ellul et al., 2020; Heming et al., 2021; Patone et al., 2021). A meta-analysis of 20 primary studies reported that among patients with COVID-19, headache, insanity, and fatigue are the most nonspecific neurological features (Mahdizade Ari et al., 2022). The pooled prevalence of neurological manifestations and mortality rate of COVID-19 patients with neurological features were estimated to be 23.0% (95% CI: 17.8–29.2) and 29.1% (95% CI: 20.3–39.8), respectively. Another meta-analysis of 19 primary studies found that the neurological symptoms of patients infected with COVID-19 mainly include fatigue, brain fog, and memory problems. Notably, the prevalence of neuropsychiatric symptoms increased significantly during medium- and long-term follow-ups. Hospitalized patients had a reduced frequency of olfactory disturbances, anxiety, depression, dysgeusia, fatigue, headache, myalgia, and sleep disturbances at 3 months (or longer) post-infection compared to non-hospitalized patients (Premraj et al., 2022).

**Figure 2:**
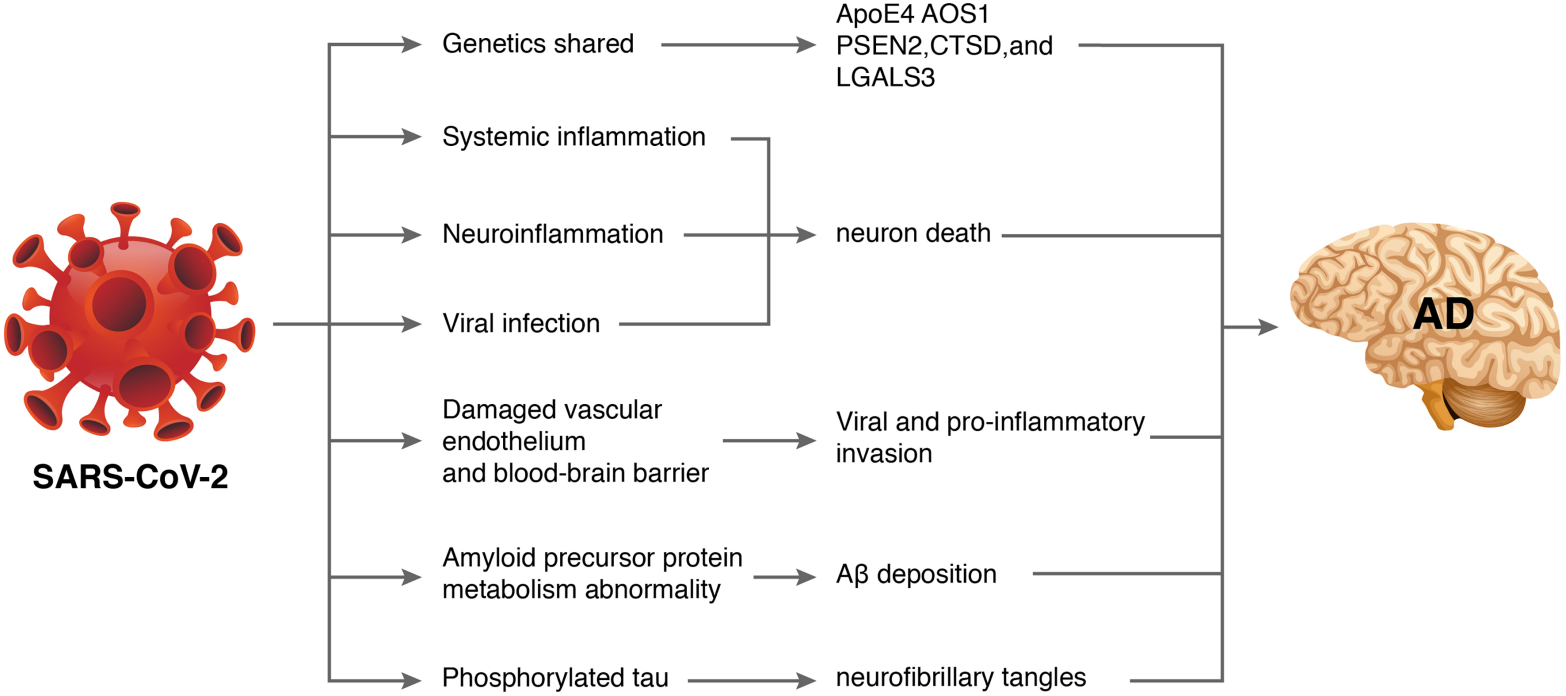
How severe acute respiratory syndrome coronavirus 2 (SARS-CoV-2) might cause Alzheimer’s disease (AD). Some database-based studies have revealed that SARS-CoV-2 and AD may share common genetic genes. Additionally, SARS-CoV-2-induced inflammatory storms lead to systemic inflammation. Entry of pro-inflammatory factors into the central nervous system through a dysfunctional blood–brain barrier induces neuroinflammation. Excessive viral replication may also penetrate the blood–brain barrier and interfere with hippocampal function leading to neurological damage and neurodegeneration. Furthermore, SARS-CoV-2 may affect amyloid precursor protein processing and phosphorylated tau leading to abnormal amyloid beta metabolism and neurofibrillary tangles.

Some patients also experienced progressive cognitive impairment, worsening of pre-existing cognitive deficits, and persistent disruptions in comprehension, reasoning, and memory (Mahase, 2021). An observational clinical study reported long-term cognitive abnormalities in nearly 50% of COVID-19 survivors (Mazza et al., 2021). A survey of 3,762 COVID-19 participants from 56 countries suggested that the most common symptoms included fatigue (98.3%), discomfort after exertion (89.1%), and cognitive impairment (85.1%) after 7 months of follow-up. Among patients whose symptoms persisted for longer than 6 months, greater than 50% developed fatigue (80%), discomfort after exertion (73.3%), and cognitive impairment (58.4%) (Davis et al., 2021). A recent meta-analysis of 27 studies showed that patients with COVID-19 had worse general cognitive function than people without COVID-19 during the acute exacerbation and assessment period 6 months after infection. Cognitive impairment is positively associated with increasing age (Readhead et al., 2018). Another meta-analysis indicated that cognitive impairments, such as brain fog, memory problems, and attention deficits, were key features of post-COVID-19 syndrome (Crivelli et al., 2022). Because AD primarily manifests as cognitive decline, whether SARS-CoV-2 increases the risk of AD has aroused widespread concern. Recent work has tried to find some clues. A study evaluating newly diagnosed dementia in the COVID-19 population found that COVID-19-positive patients had a higher incidence of dementia diagnoses and new-onset mild cognitive impairment than the general population (Kim et al., 2022). These lines of evidence support the hypothesis that brain infection with SARS-CoV-2 promotes AD-like symptoms in COVID-19 patients. Although direct evidence has not emerged, the mechanisms involved may include shared genes, systemic inflammation, neuroinflammation, vascular endothelial injury, direct viral infection, abnormal amyloid precursor protein metabolism, and phosphorylated tau (Figure 2).

### Potential mechanisms of SARS-CoV-2-induced AD

#### Shared genetics

There is growing evidence that several genetic loci are shared between AD and COVID-19, with similar directions of impact on the risk of both diseases. Magusali et al. (2021) suggested that the OAS1 rs1131454 variant was associated with increased AD risk *via* the genetic analysis of 1,313 sporadic AD patients and 1,234 controls. Notably, OAS1 was recently implicated in COVID-19 outcomes as a link for the risk of both diseases at the genetic level. Wang et al. (2022) developed a risk diagnostic model based on a common hub gene in patients with COVID-19 and AD and suggested that patients with COVID-19 are more likely to develop AD. They found four central genes (RAPGEF3, ITPKB, ITPR1, and ITPR3) as biomarkers for predicting AD in COVID-19 patients. COVID-19-mediated AD risk may be related to innate and adaptive immune responses to the virus, as demonstrated in Quincozes-Santos et al. (2021). Using SARSCOVIDB platform analysis, they found that a protease that may be involved in immune system responses, CTSL, was upregulated in brain tissue samples from 18 AD patients. The ApoE4 allele may be another risk gene for subsequent dementia risk in COVID-19 becauseApoE4 allele carriers are at high risk for AD, and it affects the severity and prognosis of COVID-19 clinical manifestations. Manzo et al. (2021) suggested that SARS-CoV-2-induced olfactory and olfactory dysfunction may confer a higher risk of dementia (specifically LOAD, DLB, and LB-variant AD) in ApoE4 carriers than in non-SARS-CoV-2-induced ApoE4 carriers. Follow-up of COVID-19 patients with ApoE4 may help identify early stages of dementia to take full advantage of any available therapy. A recent study identified differentially expressed genes associated with AD, including PSEN2, CTSD, and LGALS3, from a clinical dataset of COVID-19 patients. Notably, these genes are involved in AD pathological progression (Qiu et al., 2022). However, gene sharing only indicates the common inherited genes of the two diseases but not the direction of causality. Most of the published work is based on information from genetic databases, and data from the tissue of COVID-19 patients are lacking. Therefore, further research is needed to fill this gap.

#### Systemic inflammation

**Figure 3:**
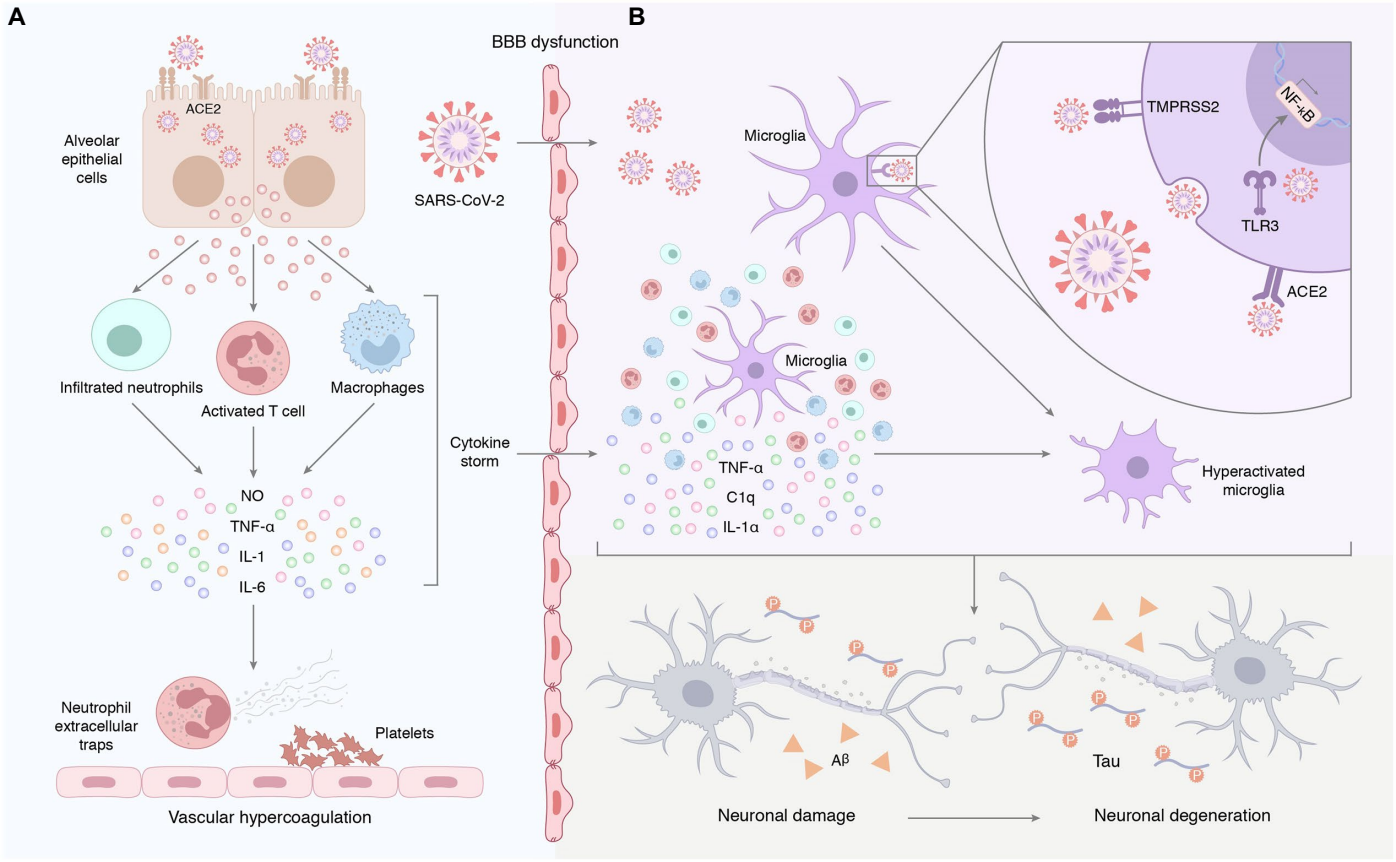
**(A)** Systemic inflammation induced by SARS-CoV-2 infection. Early unresolved viral replication may be responsible for the infiltration of infected alveolar epithelial cells, macrophages, and leukocytes into the lung tissue for overproduction of cytokines and chemokines. Therefore, we hypothesized that the neurological complications of COVID-19 may result from systemic cytokine storm and subsequent endothelial and blood–brain barrier dysfunction. **(B)** Neuroinflammation triggered by SARS-CoV-2 infection. On the one hand, peripheral pro-inflammatory factors enter the central nervous system through the damaged blood–brain barrier, prompting the hyperactivation of microglia. On the other hand, SARS-CoV-2 enters the central nervous system and binds to ACE2 receptors on the surface of glial cells to activate microglia, followed by the release of IL-1α, TNF, and the complement component C1q. Furthermore, exposure to the virus or its components promotes the expression and activation of Toll-like receptors in glial cells. This signal promotes the production and release of pro-inflammatory mediators and induces an inflammatory response in the CNS. Astrocytes can also be infected by SARS-CoV-2. The spike protein on SARS-CoV-2 virus has different receptors for astrocytes and lung cells, which enables them to bind to astrocytes. Because the infected astrocytes could not provide food for neurons, eventually leading to neuron death.

Patients with COVID-19 often exhibit severe innate immune responses and persistently elevated systemic cytokine levels. Inflammatory cytokines, such as IL-1β, IL-6, IL-12, and TNF-α, were significantly elevated in the peripheral blood of COVID-19 patients compared to healthy individuals (De Felice et al., 2020). Sun et al. (2021) found that IL-4 was elevated in the plasma cytokines of COVID-19 participants. In comparison with COVID-19 individuals without neurological manifestations, IL-6 was positively associated with sequelae severity in COVID-19 individuals with neurological manifestations (Sun et al., 2021). Systemic inflammation affects cognitive function and promotes neurodegenerative disease progression. For instance, human cognitive function is negatively correlated with the chronic peripheral elevation of IL-6 (Cojocaru et al., 2011). The increase of IL-1 in rodent brains causes long-term cognitive impairment and increased Aβ and NFT production (Rachal et al., 2001). Knockout or blocking of pro-inflammatory cytokines, such as IL-1 and IL-6 in model mice can improve their spatial memory ability and make them have better cognitive performance (Bialuk et al., 2018). Systemic inflammation may be the pathophysiological mechanism of AD pathogenesis (Akiyama et al., 2000). Compared to healthy subjects, the levels of peripheral IL-1β, IL-2, IL-6, IL-18, and high-sensitivity CRP in AD patients were up-regulated. A meta-analysis evaluating the association between peripheral IL-6 and CRP in healthy adults found that higher concentrations of inflammatory markers increased the risk of AD. TNF-α converting enzyme, soluble TNF receptors 1 and 2, α1-antichymotrypsin were increased, and IL-1 receptor antagonism in the peripheral blood decreased. Systemic inflammation characterized by a ‘cytokine storm’ in severe cases of COVID-19 disrupts the blood–brain barrier and induces neuronal and glial cell damage (Mohammadi et al., 2020). Virus-induced systemic inflammatory storms, coupled with increased blood–brain barrier (BBB) permeability, lead to the entry of a large number of mediators into the central nervous system, which amplifies neuroinflammation and promotes neurodegenerative processes (Maes et al., 2022). Astrocytes can also be infected by SARS-CoV-2. Astrocytes exist in the brain. They make and transport food for neurons. The spike protein on SARS-CoV-2 virus has different receptors for astrocytes and lung cells, which enables them to bind to astrocytes. Because the infected astrocytes could not provide food for neurons, eventually leading to neuron death (Crunfli et al., 2022; Figure 3A). Peripheral inflammatory factors also circulate to the brain and activate cerebral microglia and astrocytes, which secrete pro-inflammatory cytokines that amplify the effects of neuroinflammation (Wang et al., 2019; Kucia et al., 2021).

#### Neuroinflammation

**Figure 4:**
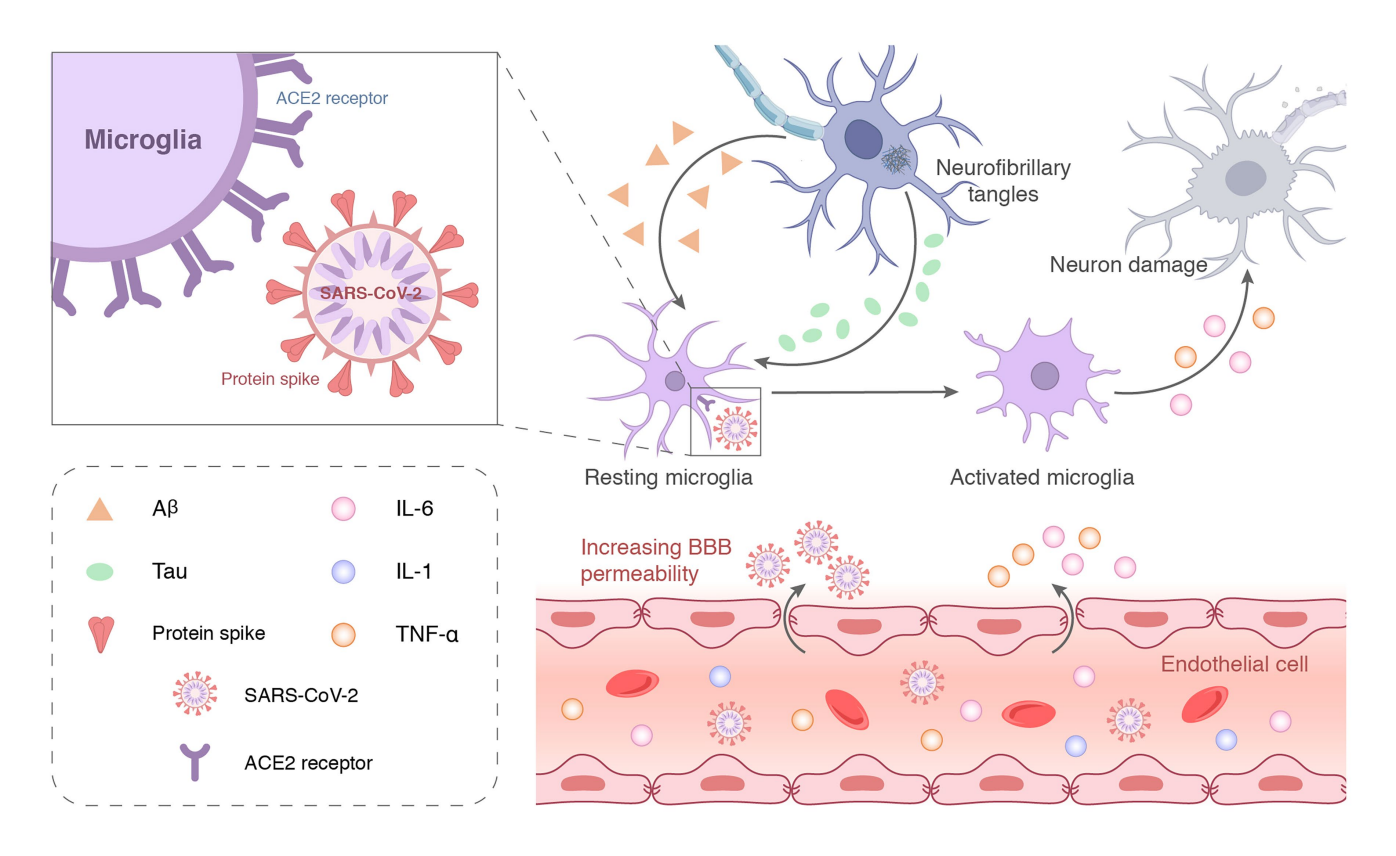
SARS-CoV-2 can enter the central nervous system through the damaged blood–brain barrier and bind to ACE2 receptors on the surface of microglia to activate the immune function. The large-scale replication of the virus over-activates microglia or induces chronic stimulation by long-term latency, which eventually leads to neuronal incapacitation and damage.

Increasing evidence emphasizes the contribution of neuroinflammation to AD, which likely plays a vital role in the early stages of the disease where intervention may be most beneficial. Although Aβ deposition is the initiating step of AD pathology, neuroinflammation may be the main driving force of neurodegeneration (Park et al., 2018; Desforges et al., 2020). Neuroinflammation may be the entry of proinflammatory factors into the brain through the damaged BBB or the direct intrusion of SARS-CoV-2 to induce an inflammatory response (Figure 3B). COVID-19-mediated neuroinflammation is manifested by the activation of microglia and astrocytes. SARS-CoV-2 reaches the olfactory bulb of the hypothalamus through the neuroepithelium of the olfactory mucosa (Steardo et al., 2020). The presence of SARS-CoV-2 in the olfactory bulb may lead to the activation of mast cells, microglia, and astrocytes, which leads to the tissue release of pro-inflammatory cytokines (Rutkai et al., 2022). Brains infected with SARS-CoV-2 exhibited a marked inflammatory response and extensive activation of microglia and astrocytes compared to brains without SARS-CoV-2 (Rutkai et al., 2022). Data from an *in vitro* study highlighted that the SARS-CoV-2 spike protein S1 increased the release of TNF-α, IL-6, IL-1β, and iNOS/NO by stimulating BV-2 microglia (Olajide et al., 2022). Evidence from *in vitro* and *in vivo* studies is complemented by clinical samples. Ziff et al. (2022) found that pro-inflammatory cytokines, such as TNFα, IL6, IL1β, and IL8, were significantly increased in the cerebrospinal fluid of patients with COVID-19 neurological syndrome. Poloni et al. (2021) found that microglial activation in the brainstem was significantly increased in COVID-19 cases, and abnormal microglial activation in the frontal cortex and hippocampus was associated with AD pathology. Notably, hippocampal microglia was significantly enhanced in COVID-19 patients with delirium (all patients with neurocognitive impairment). However, no traces of SARS-CoV-2 colonization were found in brain tissue. Schwabenland et al. (2021) detected profound immune activation in the brain tissue of deceased patients with COVID-19, which was manifested by specific CD8 T-cell clusters affecting the vasculature and prominent CD8 T-cell-microglia crosstalk in the parenchyma. This study highlights that SARS-CoV-2 is recognized in cerebrovascular structures and provides a possible basis for broad immune activation. This severe neuroinflammation may partially explain the underlying mechanism of SARS-CoV-2 infection-induced AD development. As innate immune cells in the brain, microglia have long been considered to be related to the pathology of neurodegenerative diseases. More and more shreds of evidence show that activated microglia can produce a variety of neurotoxic factors including pro-inflammatory mediators and reactive oxygen species for a long time, thus causing persistent damage to neuronal. Microglia can be activated for a long time due to single stimulation (e.g., pathogen infection) or multiple stimulations, to aggravate the damage of neurons over time. Brain-targeted SARS-CoV-2 infection can induce microglia activation, then cause chronic inflammation and eventually lead to neurodegeneration (Figure 4). Moreover, neuroinflammation is associated with intense oxidative stress that exacerbates neurodegenerative diseases, such as AD. COVID-19 patients with advanced age and comorbidities with an inflammatory basis (e.g., diabetes, atherosclerosis, and subclinical dementia) may be at increased risk of AD. Therefore, neuroinflammation caused by COVID-19 may be unmanageable, especially in older adults, because their immune system responds less efficiently (Kritas et al., 2020; Schirinzi et al., 2021).

#### Viral infection

Angiotensin-converting enzyme 2 (ACE2) receptor is the primary mediator of SARS-CoV-2 entry into cells. Some studies suggest that SARS-CoV-2 may be “neuroinvasive” and directly infect neurons or glial cells in the central nervous system (Figure 4). These studies, without exception, found ACE2 expressed in neurons of the brain. SARS-CoV-2 was identified throughout the brain parenchyma in one autopsy sample using antigen staining (Xu et al., 2005). Two dissertations demonstrated ACE2 staining of astrocytes present in the cerebellum and brainstem of rats (Gallagher et al., 2006; Gowrisankar and Clark, 2016). According to a meta-analysis of single single-cell sequencing data sets, ACE2 is expressed at low levels in the human posterior cingulate cortex and middle temporal gyrus (Chen et al., 2020). It is worth noting that SARS-CoV-2 may infect cortical neurons in human brain organoids (which express ACE2) (Bilinska et al., 2020). Knock human ACE2 gene into mouse ACE2 locus, and then intranasal drip of SARS-CoV-2 causes neuronal infection in the brain, but the specific number and distribution of these infected cells have not been determined (Sun et al., 2020). These studies highlight that the expression of ACE2 receptors in brain neurons facilitates SARS-CoV-2 invasion. The presence of SARS-CoV-2 in the brains of COVID-19 patients supports this hypothesis (Matschke et al., 2020; Puelles et al., 2020). Serrano et al. (2021) performed RT-PCR of 16 brain regions in 20 subjects who died of COVID-19 and found that most subjects had non-specific histopathology, including focal β-amyloid precursor protein immunoreactivity and sparse perivascular mononuclear cell cuffs. SARS-CoV-2 RNA was detected in one or more brain regions of four subjects, including the olfactory bulb, amygdala, intra-olfactory region, temporal and frontal neocortex, dorsal medulla, and pia mater (Serrano et al., 2021). An autopsy report on 44 patients who died of COVID-19 has attracted attention. These researchers systematically examined viral load in multiple organs throughout the body, including the brain. The spread characteristics and different damage to various organs after virus infection were studied. A total of 85 anatomical locations and 79 types of body fluid samples were involved, and SARS-CoV-2 RNA detected was detected in all samples. After detection, SARS-CoV-2 RNA was found in 90.9% of brain tissues, second only to lung tissue. This study is the first report to directly demonstrate that SARS-CoV-2 spread in multiple organs and the brain early in infection, and multiple extrapulmonary replication sites were preserved during the first week after symptom onset. These extrapulmonary tissues are likely to prolong the replication time of the virus and lay the foundation for future complications, especially in the nervous system. A recent large study comparing brain scans of the same person before and after infection with SARS-CoV-2 found that brain changes may be an inevitable outcome of SARS-CoV-2 invasion (Douaud et al., 2022). This study also found that participants had more gray matter loss and tissue abnormalities a few months after SARS-CoV-2 infection, primarily in brain regions involved in smell than participants who were not infected with SARS-CoV-2. Some of the altered areas of the brain identified in the study are also involved in memory-related functions.

Once SARS-CoV-2 enters the brain, it binds to ACE2 receptors on neurons, astrocytes, oligodendrocytes, and microglia. Their interactions may lead to astrogliosis and microgliosis, increase BBB permeability, and allow monocytes and leukocytes to infiltrate multiple brain regions of the central nervous system (Vargas et al., 2020), including the olfactory bulb, choroid plexus, cerebral cortex, and middle temporal gyrus (Chen R. et al., 2020; Chen L. et al., 2020; Figure 4). Due to viral neurotropism, SARS-CoV-2 spreads through neuroanatomically interconnected pathways and causes neuronal dysfunction and neurodegeneration in the central nervous system.

#### Damaged blood–brain barrier

The systemic inflammation induced by COVID-19 may lead to rupture of the BBB, which promotes immune cell infiltration (Hascup and Hascup, 2020; Figure 3A). Cytokine storms in the brain also cause rupture of the BBB, which leads to a vicious cycle of increased pathology (Najjar et al., 2020). Therefore, we hypothesized that systemic cytokine storms and subsequent endothelial and BBB dysfunction in COVID-19 patients will increase the risk of AD in subsequent decades, especially in elderly individuals, because the gradual loss of blood–brain barrier integrity is an aging characteristic. However, the length of SARS-CoV-2-mediated disruption of the vascular endothelium and BBB persists is not clear but is the focus of future research.

#### Amyloid precursor protein metabolism abnormality

The major event in AD is the uncontrolled accumulation of toxic Aβ due to an imbalance in production and removal. The core pathway leading to the accumulation of Aβ involves cleavage of the amyloid precursor protein (APP) by γ-secretase. Notably, the multi-group analysis of COVID-19 patient samples shows that there is a potential relationship between the metabolic process of APP and COVID-19 infection. Caradonna et al. (2022) found that the expression of APP was up-regulated after the SARS-CoV-2 virus was bound to the ACE2 receptor. This result is consistent with Camacho et al. (2021) who identified 6 upstream regulators that increased APP production in a COVID-19 patient dataset. RNA-seq analysis highlighted that patients with COVID-19 had significantly increased APP transcripts in blood samples compared to those without COVID-19 (Overmyer et al., 2021). Derived from a study on single-cell RNA-seq by Yang et al., oligodendrocytes were isolated from the brain tissue of dead COVID-19 patients. It was found that APP was one of the most up-regulated genes (Yang et al., 2021). IFITM3 is a viral restriction protein that sequesters viral particles and subsequently transports them to lysosomes. Vavougios et al. (2021) demonstrated dysregulation of IFITM3-dependent pathways in neurons and peripheral immune cells donated from AD patients and this perturbation may be induced by a variety of viruses, including SARS-CoV-2. Notably, IFITM3 upregulation induced γ-secretase activity, which increased amyloid production (Idrees and Kumar, 2021). The heparin-binding site on the S1 protein may help amyloid protein bind to the virus surface, which triggers the aggregation of these proteins and ultimately led to neurodegeneration in the brain. However, there are some inconsistent arguments. For example, multiple transcriptome sequencing of whole blood with red blood cells removed failed to identify differences in the expression levels of APP and PSEN1/2 between COVID-19-infected and uninfected individuals (Tang et al., 2020). There was no significant difference in the transcriptional levels of APP and PSEN1/2 in brain organs derived from pluripotent stem cells between SARS-CoV-2 infected and uninfected persons (Jacob et al., 2020). Therefore, the effect of COVID-19 on APP metabolism still needs further exploration.

#### Phosphorylated tau

Neurofibrillary tangles formed by hyperphosphorylated tau protein are another important neuropathological marker of AD. Recent study suggested that the plasma T-tau concentration of COVID-19 patients without obvious neurological symptoms is significantly higher than that of COVID-19 negative patients, indicating that SARS-CoV-2 may cause tau protein hyperphosphorylation (Lennol et al., 2023). Although there is no evidence to prove the causal relationship, the highly expressed ACE2 receptor in the brain provides a potential mechanism, because the high expression of ACE2 receptor in the brain was inversely proportional to the cognitive decline of AD patients, and was positively correlated with insoluble phosphorylated tau (Louise et al., 2023).

## Discussion

Infectious agents, especially viruses, are likely to be involved in the pathogenesis of AD. According to this hypothesis, these viral particles evade the host immune system and lead to chronic infection and the subsequent deposition of Aβ and phosphorylated tau in the brain. Although several AD-related candidate viruses were identified in previous studies, the specific mechanisms remain obscure. These studies are also only correlational rather than causative. The validation of this hypothesis has not stopped because therapeutic strategies targeting Aβ are being questioned. The frequent neurological manifestations of SARS-CoV-2 have forced researchers to consider the explosion of neurodegenerative diseases in the ensuing decades. Several studies have observed cognitive impairment in COVID-19-infected people, which suggests that COVID-19 plays a role in the development of AD. However, there is currently no direct evidence that the neuropathology of COVID-19 is caused by the direct viral infection of the central nervous system or the accompanying immune response and the resulting hypercoagulable state.

Some researchers suggest a more reserved response. They concluded that although viral RNA is detected in approximately 50% of cases, only small amounts of viral protein are present in isolated neurons and endothelial cells in the medulla oblongata of the central nervous system (Matschke et al., 2020; Meinhardt et al., 2021). These low levels of expression are not sufficient to cause neurodegeneration. The severity of neuropathy also did not correlate with the presence of viral proteins or RNA in the brain (Matschke et al., 2020). SARS-CoV-2 may not possess neuro-invasive activity because the ACE2 receptor is only rarely expressed or almost absent from neuronal cells (Brann et al., 2020). However, the presence of the virus may be influenced by the time interval between initial infection, death, autopsy, and subsequent brain processing. Notably, the expression of ACE2 is closely related to the specific detection method used. For example, single-cell sequencing techniques have low sensitivity, and immunohistochemistry depends on the anti-ACE2 antibody. Conclusions based on animal and cellular models of SARS-CoV-2 infection depend on the expression pattern of the ACE2 protein and S protein affinity in each model, which may affect the necropsy results (Cooper et al., 2020).

### Directions for future research

Addressing these issues may require further research. First, preclinical studies may be performed immediately. Various AD animal models were used to examine key pathological outcomes of SARS-CoV-2 infection, including amyloid plaques, cognitive function, and activation of immune cells in the brain. Second, there are few studies on AD-related pathological changes in post-mortem tissue, especially brain tissue, of COVID-19 patients, and the sample size is limited. This hinders our exploration of the potential link between COVID-19 and AD. Thus, more detailed classification and larger sample size research are necessary, such as focusing on patients who have a longer infection or are more ill. Third, long-term complications of COVID-19 are expected to emerge over the next 10–20 years. Therefore, assessing the risk of long-term neurological sequelae of COVID-19 will be critical, especially in elderly and severely ill patients. With the increasing number of COVID-19-infected people, it is necessary to establish a long-term follow-up system for them because many important retrospective studies will prove whether COVID-19 survivors have a greater risk of AD. Notably, the prospective cohort study design will help us better understand the subsequent AD risk of COVID-19. Fourth, a global registry system is urgently needed to collect clinical data regularly and help researchers analyze participants’ cognitive functions to better understand the occurrence, natural history, and optimal management of COVID-19-related AD. By creating a global registry, scientists around the world are called to work together to quickly share relevant clinical observations and help answer the above questions.

## Conclusion

Extensive research over the past few decades suggests that viral infections are involved in the etiology of AD. Antiviral therapy may be a promising target for modulating AD. The SARS-CoV-2 virus that has infected hundreds of millions of people seems to show no signs of stopping after 3 years of rage. Neuroscientists must consider long-term sequelae. Despite growing knowledge about the potential of SARS-CoV-2 in causing AD, there is no consensus on specific contributions. However, it is important to highlight this possibility and act now to prepare for an unknown future.

## Author contributions

HL and XJ: conceptualization. NL: investigation and resources. NL and XJ: writing—original draft preparation. XJ: writing—review and editing. HL: visualization and supervision. All authors contributed to the article and approved the submitted version.

## Funding

This work was supported by the China Academy of Chinese Medical Sciences Innovation Fund (Nos. CI2021A01401 and CI2021A01405).

## Conflict of interest

The authors declare that the research was conducted in the absence of any commercial or financial relationships that could be construed as a potential conflict of interest.

## Publisher’s note

All claims expressed in this article are solely those of the authors and do not necessarily represent those of their affiliated organizations, or those of the publisher, the editors and the reviewers. Any product that may be evaluated in this article, or claim that may be made by its manufacturer, is not guaranteed or endorsed by the publisher.
